# Atypical sensory processing features in children with autism, and their relationships with maladaptive behaviors and caregiver strain

**DOI:** 10.1002/aur.2700

**Published:** 2022-03-17

**Authors:** Zoe A. M. Griffin, Kelsie A. Boulton, Rinku Thapa, Marilena M. DeMayo, Zahava Ambarchi, Emma Thomas, Izabella Pokorski, Ian B. Hickie, Adam J. Guastella

**Affiliations:** ^1^ Autism Clinic for Translational Research, Brain and Mind Centre, Children's Hospital Westmead Clinical School, Faculty of Medicine and Health University of Sydney Camperdown Australia

**Keywords:** autism spectrum disorder, caregiver strain, maladaptive behaviors, pediatric, sensory processing

## Abstract

Atypical sensory processing occurs in up to 97% of children on the autism spectrum. Children who are on the autism spectrum also commonly demonstrate challenging behaviors, and their caregivers report increased levels of strain in daily life. The aim of this study was to explore four sensory processing features; seeking, avoiding, sensitivity, and registration, and their relationships with maladaptive behaviors in children with autism, as well as with caregiver strain. Participants comprised 75 children with autism aged 7–12 years (*M* = 7.81). Caregivers completed three questionnaire measures examining child sensory processing, maladaptive behaviors, and perceptions of caregiver strain. We found avoiding significantly associated with irritability. Avoiding also displayed the strongest relationship with global caregiver strain. Avoiding and seeking were strongly related to hyperactivity/noncompliance (components of maladaptive behavior). A multiple regression was performed to explore how atypical sensory processing features and maladaptive behaviors together predicted caregiver strain. Together, maladaptive behaviors and sensory features accounted for 58% of the variance in total caregiver strain. The only significant individual predictor of total caregiver strain was sensory avoiding, which uniquely accounted for 5.76% of the variation. The findings suggest that atypical sensory processing is associated with overall caregiver strain, above that explained by maladaptive behaviors. Implications for targeted support for the benefit of the child, parents and family unit are discussed.

**Lay summary:**

Children who are on the autism spectrum often have differences in sensory processing. These children also tend to show challenging behaviors, and their caregivers can experience increased stress. This study looked at how sensory processing difficulties relate to such behaviors and caregiver stress. We found that both sensory processing and challenging behaviors were related to the amount of stress caregivers felt. This suggests that interventions may benefit from looking at sensory processing features when considering how to help reduce challenging behaviors and caregiver stress.

## INTRODUCTION

Atypical sensory processing is a common feature of autism. It is reported from early in development, with up to 97% of children with autism displaying features of atypical sensory processing that persists across the lifespan (Dellapiazza et al., 2018). Such sensory processing atypicality refers to challenges regulating the type and intensity of behavioral responses to sensory input (Miller et al., 2007). For example, some individuals may be distressed when faced with lights, loud sounds, or particular tactile sensations. To reflect this, the most recent version of the Diagnostic and Statistical Manual of Mental Disorders (DSM‐5) features sensory issues within the diagnostic criteria for autism (though not essential for diagnosis) (American Psychiatric Association, 2013). There is a plethora of research examining the impact of atypical sensory processing on adaptive functioning, though relatively little attention is given to its relationship with maladaptive behaviors (such as irritability, aggression or withdrawal), or with caregiver strain (i.e., the perceived negative effects of caring for a child with different needs). Given that children on the spectrum demonstrate increased rates of maladaptive behaviors (Brereton et al., 2006), and caregivers of children with autism are likely to report elevated strain (Kirby et al., 2015), a clearer understanding of the relationships between atypical sensory processing, maladaptive behaviors, and caregiver strain is warranted. Such an investigation has important implications for social and educational outcomes in children who have autism, and the psychological wellbeing of their caregivers and families.

There is considerable heterogeneity in the sensory features of individuals with autism (Uljarević et al., 2017). To categorize these features, Dunn (2014) refined her model of atypical sensory processing consisting of four sensory features, or “quadrants”: seeking (searching for intense stimulation), avoiding (escaping sensory stimulation), sensitivity (intense and usually negative behavioral response to an objectively nonthreatening stimulus), and poorer registration (reduced or absent response to a sensory stimulus). Individuals with autism can display varying combinations of these features, with differing levels of severity (Hand et al., 2017; Koenig & Kinnealey, 2008). Recent evidence suggests there may be sex differences in sensory processing features, with females showing increased atypicality relative to males with autism (Osório et al., 2021).

Several studies in children with autism have observed positive relationships between the strength of atypical sensory features and maladaptive behaviors (Baker et al., 2008; Nieto et al., 2017). Maladaptive behaviors are typically defined as disruptive, destructive, or aggressive behaviors as well as irritability, lethargy, or hyperactivity (Aman et al., 1985; Dominick et al., 2007). Such behaviors within autism can include harm to others (biting, kicking), self‐injurious behaviors (head banging, biting, or hitting oneself), and severe tantrums (Dominick et al., 2007; Fulton et al., 2014). Naturally, such behaviors pose a challenge to children across many domains, particularly with respect to social participation and establishing social relationships (Fulton et al., 2014; Koenig & Kinnealey, 2008). These behaviors can interfere with learning in educational settings (Horner et al., 2002).

Select studies have investigated distinct relationships between different atypical sensory features and maladaptive behaviors in children with autism. For instance, atypical sensory processing appears to explain a considerable amount of variance in such behaviors, after controlling for age and intellectual level (Dellapiazza et al., 2020). Notably, across studies increased sensory avoiding behaviors have been associated with increased irritability (Dellapiazza et al., 2020), while sensory seeking and sensitivity behaviors may be related to hyperactivity and antisocial behaviors (Baker et al., 2008; Dellapiazza et al., 2020). Other findings provide support for a relationship between atypical sensory features, such as avoiding and sensitivity, and internalizing and externalizing problems more broadly in children on the autism spectrum (Tseng et al., 2011).

Taken together, findings to date provide strong evidence for differential links between sensory processing and maladaptive behaviors. However, many prior studies in this area have not utilized measures validated specifically for assessing maladaptive behaviors in a population of individuals with autism. Instead, many studies have employed the optional supplementary “maladaptive behavior” section of the Vineland Adaptive Behavior Scales, second edition (VABS‐II; Sparrow et al., 2005). The VABS‐II was created to assess adaptive behavior, and the maladaptive behavior section provides only a brief assessment of internalizing and externalizing behaviors. While the VABS‐II is a well validated measure of adaptive behavior within populations of children with autism, the use of the measure in assessing maladaptive behaviors in this population has been questioned (Weiss et al., 2010). Dellapiazza et al. (2020) noted that their study was among the first to utilize an instrument validated to measure maladaptive behaviors in individuals with autism (namely, the Aberrant Behavior Checklist; ABC) when exploring their relationship with sensory features. Thus, there is a need to examine the role of sensory features in maladaptive behaviors using an instrument which has been validated to do so for this population.

For individuals who are on the autism spectrum, the severity of maladaptive behaviors is also associated with the degree of caregiver strain (e.g., see Davis & Carter, 2008). High levels of strain have been shown to be a significant risk factor for depression in caregivers of children who are on the spectrum (Abbeduto et al., 2004; Bromley et al., 2004). Furthermore, research has indicated that caregiver stress can negatively influence parenting behaviors, such as providing less responsiveness or warmth when interacting with children, or harsh discipline, increasing the risk of a child developing anxiety or displaying aggressive behavior (Chiang et al., 2019; Davis & Carter, 2008; Hall & Graff, 2012). Although the relationships between atypical sensory processing and maladaptive behaviors, and between maladaptive behaviors and caregiver strain, have been investigated separately, there is limited research investigating the relationship between these three domains.

Recent studies by Chiang et al. (2019) and Kirby et al. (2019) found that caregivers whose children exhibited increased atypicality in sensory processing reported increased parenting stress. However, preliminary research examining caregiver burden varying by sensory features or subtypes has found inconsistent results. In part, this has been due to the terminology and characterization of sensory patterns which vary across measures utilizing different conceptual models of sensory processing (Chen, 2021). For example, atypical scores on “Tactile Sensitivity” and Auditory Processing have been associated with poorer caregiver mental health (Suzuki et al., 2019). Higher levels of maternal strain have been associated with elevated sensitivity (Ben‐Sasson et al., 2013) and elevated avoiding scores (Ben‐Sasson et al., 2013; Nieto et al., 2017). Additionally, Kirby et al. (2015) demonstrated that over and under‐sensory reactions, and unusual interest in sensations, predicted caregiver strain in caregivers of children with autism (but, interestingly, not in caregivers of children with other developmental disabilities). Hand et al. (2018) found caregiver strain was significantly associated with deficits in either sensory reactivity or multisensory integration. While terminology differs across studies with respect to sensory processing features, taken together the literature suggests that increased levels of atypical sensory processing (particularly avoidance and sensitivity) are related to increased caregiver strain.

Against this background and using the four sensory quadrants identified by Dunn (1997), the current study investigated the relationships between atypical sensory processing features, maladaptive behaviors, and caregiver strain in children with autism. Our first aim was to investigate differential relationships between sensory features and maladaptive behaviors in children with autism, using an established measure of maladaptive behavior, namely, the aberrant behavior checklist (ABC‐P; Aman et al., 1985). Given the findings of Dellapiazza et al. (2020) who utilized this same measure, we anticipated sensory avoiding behaviors would relate to increased irritability, while sensory seeking and sensitivity behaviors would be related to increased hyperactivity and noncompliance. Our second aim was to consider the relationship between sensory features and the degree of caregiver strain. Given the variability of findings of previous studies, we could not make any specific hypothesis regarding differential relationships. However, we hypothesized that increased atypical sensory processing would be associated with elevated caregiver strain. The final aim of this study explored how maladaptive behaviors, and atypical sensory processing features together predict caregiver strain, and examined the individual contribution of each sensory processing quadrant to this relationship. While we could not make any specific hypotheses based on the extant literature, we anticipated that sensory processing atypicality would account for some of the variances in the relationship between maladaptive behaviors and caregiver strain, given the findings described above (Ben‐Sasson et al., 2013; Nieto et al., 2017).

## METHOD

### Participants

A total of 75 children (65 males, 10 females) with autism aged between 7 and 12 years (M = 7.81, *SD* = 2.61) took part in the study, conducted at the Brain and Mind Centre at The University of Sydney. A diagnosis of autism was confirmed using the autism diagnostic observation schedule‐2nd edition (ADOS‐2; Lord et al., 2012), administered by research‐reliable assessors, and a clinical interview assessing DSM‐5 criteria. Caregivers provided written, informed consent prior to taking part in the study, and the study was approved by the University of Sydney Human Research Ethics Committee (HREC; Ref no: 2013/502).

### Measures

#### Autism diagnosis

The ADOS‐2 (Lord et al., 2012) was used to confirm a diagnosis of Autism Spectrum. This semi‐structured, standardized assessment presents various activities that elicit behaviors directly related to a diagnosis of autism. Module 1 is used for children 31 months and older who do not consistently use phrase speech, Module 2 is used with children of any age who are not verbally fluent but do use phrase speech, and Module 3 is used for verbally fluent children and younger adolescents. Scores can be converted to calibrated severity scores (CSSs) to allow comparison across different modules (Gotham et al., 2009; Hus et al., 2014). These CSSs provide a total score, a social affect score, and restricted and repetitive behavior score (Gotham et al., 2009). CSSs range from 0 to 10 and enable comparison of symptom severity across different modules, with higher CSSs corresponding to higher symptom severity. High interrater and test–retest reliability, as well as high validity, has been previously established (Chojnicka & Pisula, 2017; Lord et al., 2012).

#### IQ estimate

An estimate of non‐verbal intellectual functioning was obtained using the Leiter international performance scale‐third edition (Leiter‐3; Roid & Miller, 1997). The Leiter‐3 Nonverbal IQ composite score was used as the measure of nonverbal IQ in this study. The Nonverbal IQ composite is a standard score (M = 100; *SD* = 15) derived from subtest scores that measure nonverbal intelligence by assessing problem‐solving and logical reasoning using visualization (e.g., pattern recognition, visual recognition, visual closure). This non‐verbal measure of intelligence was designed for individuals aged from 3 to 75 years and has been validated in children with autism (Kasari et al., 2014; Tsatsanis et al., 2003).

#### Sensory processing

Sensory processing was assessed using the Short Sensory Profile‐2 (SSP‐2), a 34‐item scale which measures the extent and severity of sensory processing impairments and associated behaviors in children with autism (Dunn, 2014). Caregivers rate a child's typical response to sensory stimuli on a 5‐point Likert scale ranging from 1, “almost never” to 5, “almost always,” with higher scores indicating greater sensory processing difficulties. The SSP‐2 produces four domains, or quadrants: seeking, avoiding, sensitivity and registration. Additionally, it provides two summary measures: a sensory processing subscale and a behavioral responses subscale. The scale was developed for children aged 3 to 15 years. Cronbach's *α* were calculated for this sample for each of the four quadrants. Seeking consisted of 7 items (*α* = 0.79), avoiding consisted of 9 items (*α* = 0.84), sensitivity consisted of 10 items (*α* = 0.79) and registration consisted of 8 items (*α* = 0.81). Cronbach's *α* values for the sensory processing and behavioral responses subscales were also strong (*α* = 0.88 and 0.90, respectively). The measure has been found to have acceptable reliability and validity in previous research (Dunn, 2014).

#### Maladaptive behavior

Maladaptive behavior was measured using the ABC‐P (Aman et al., 1985). While this rating scale was originally developed to measure treatment effects, it is now commonly used to assess maladaptive behaviors in children with various neurodevelopmental conditions, including autism (Dellapiazza et al., 2020). The scale contains 58 items related to maladaptive behaviors, with each item scored on a 4‐point Likert scale from 0, “no problem” to 3, “severe problem.” The ABC‐P generates five subscale scores; irritability, social withdrawal, stereotypic behavior; hyperactivity/noncompliance; and inappropriate speech. The ABC‐P has strong internal consistency, test–retest reliability, and inter‐rater reliability (Aman et al., 1985). Cronbach's *α* values in the current sample were acceptable, ranging from 0.79 to 0.95 across domains.

#### Caregiver Strain

The caregiver strain questionnaire (CGSQ; Brannan et al., 1997) was used to assess caregiver strain. This self‐report questionnaire containing 21 items was originally developed to measure levels of strain related to caring for children and adolescents with emotional and behavioral disorders (Brannan et al., 1997). In addition to producing a global score, the CGSQ yields scores for three subscales: objective strain (e.g., financial impact), subjective internalized strain (e.g., sadness about the child's problems), and subjective externalized strain (e.g., anger directed toward the child). Scores are endorsed on a Likert Scale from 1 to 5, with higher scores indicating higher levels of strain (except for one reverse‐coded item). In a validation study assessing caregivers with children with autism, the 21‐item scale has been found to have excellent internal consistency, as well as satisfactory convergent and factorial validity (Khanna et al., 2012). Cronbach's *α* values in the current sample were acceptable, ranging from 0.73 to 0.94 across subscales and 0.94 for the global score.

### Data analysis

Data were analyzed using the statistical package for the social sciences (SPSS) (version 24) program. Shapiro–Wilk tests indicated that some variables deviated significantly from normality (*p* <0.05, see Table 1). Consequently, all analyses were conducted using bootstrapping (2000 resamples).

**TABLE 1:** Descriptive statistics for the SSP‐2, ABC, CGSQ, ADOS‐2, and Leiter‐3

	Range	Mean	*SD*	Shapiro Wilk
Measure				
SSP‐2				
Sensory avoiding	8–43	26.65	8.25	0.98
Sensory seeking	1–33	17.37	7.37	0.98
Sensory sensitivity	7–47	29.49	8.46	0.99
Low registration	3–37	18.17	7.99	0.98
ABC‐P				
Irritability, agitation, crying	0–37	12.19	8.90	0.94*
Lethargy/social withdrawal	0–33	10.85	7.47	0.94*
Stereotypic behavior	0–16	5.09	3.89	0.92**
Hyperactivity, noncompliance	1–45	18.31	11.12	0.96*
Inappropriate speech	0–11	4.09	3.15	0.94*
CGSQ				
Objective strain	11–50	30.68	11.54	0.95*
Subjective internalized strain	7–30	20.39	5.37	0.90**
Subjective externalized strain	4–17	7.71	3.17	0.97
Global score	24–92	58.77	17.69	0.97
Leiter‐3				
Full‐scale IQ	56–141	97.36	17.09	0.98
ADOS‐2				
CSS total	3–10	7.18	1.53	0.92**
CSS social affect	2–10	6.97	1.86	0.95*
CSS restricted and repetitive behaviors	1–10	7.29	2.32	0.86**

Abbreviations: ABC–P, aberrant behavior checklist–parent; ADOS‐2, autism diagnostic observation schedule, 2nd edition; CGSQ, caregiver strain questionnaire; CSS, calibrated severity score; SSP‐2, short sensory profile–2.

Descriptive statistics were calculated to characterize the sample and study variables. To examine the association between atypical sensory processing features and maladaptive behaviors, Pearson's correlations were used to determine the relationship between each sensory quadrant of the SSP‐2 and the subscales of the ABC‐P. To determine the relationships between differing atypical sensory processing features and caregiver strain, Pearson's correlations were performed between scores on each sensory quadrant of the SSP‐2 and the three sub‐scales as well as total score of the CGSQ. To aid in the interpretation of analyses, effect sizes are reported throughout the results, using accepted cut‐offs of 0.1 (small effect), 0.3 (medium effect) and 0.5 (large effect) (Cohen, 1988, 1992).

Finally, to examine the relationship between maladaptive behaviors and caregiver strain after accounting for atypical sensory processing in children with autism, a 2‐step hierarchal multiple regression analysis was performed.

## RESULTS

Descriptive statistics for the SSP‐2, ABC‐P, CGSQ, ADOS‐2, and Leiter‐3 are presented in Table 1. To determine if sensory processing difficulties differed between males and females, Mann–Whitney tests were conducted. Mann–Whitney tests indicated that there was no statistically significant difference in the median scores between females and males for sensory seeking (*U* = 283, *p* = 0.512), avoiding (*U* = 314, *p* = 0.864), sensitivity (*U* = 270, *p* = 0.391), or registration (*U* = 322, *p* = 0.963). Bivariate correlations were conducted to examine relationships between demographic variables (age, IQ) and our variables of interest (sensory processing, caregiver strain, maladaptive behaviors). There were no statistically significant associations observed between age or IQ and our variables of interest (*p* > 0.074, see Table S1). As such, we did not control for age or IQ in subsequent analyses.

### Associations between sensory processing and maladaptive behaviors

The first aim of the study was to determine whether the four sensory quadrants were differentially associated with maladaptive behaviors. To achieve this, Pearson correlations were performed between the SSP‐2 quadrants and domains of the ABC‐P. Table 2 demonstrates the relationship between the four sensory quadrants, and the individual domain scores of the ABC‐P. All four sensory quadrants were significantly associated with the irritability domain of the ABC‐P. Specifically, higher avoiding scores were strongly correlated with increased irritability (*r* = 0.70, *p* <0.001), while seeking (*r =* 0.44, *p* <0.001) and sensitivity (*r =* 0.46, *p* <0.001) were moderately correlated with irritability. Elevated registration scores (i.e., poor registration, reflecting hypo‐reactivity) were weakly associated with irritability (*r* = 0.29, *p* <0.05). Seeking, avoiding and sensitivity quadrants displayed significant and strong positive associations with hyperactivity/noncompliance (*r =* 0.60, *r =* 0.60, and *r =* 0.54 respectively, *p* <0.001). Poorer registration was also significantly, though moderately, associated with hyperactivity/noncompliance (*r =* 0.41, *p* <0.001).

**TABLE 2:** Pearson's correlations between sensory quadrants and maladaptive behaviors

	Seeking	Avoiding	Sensitivity	Registration
Irritability, agitation, crying	0.44**	0.70**	0.46**	0.29*
Lethargy/social withdrawal	0.41**	0.48**	0.58**	0.34*
Stereotypic behavior	0.42**	0.29*	0.43**	0.26*
Hyperactivity, noncompliance	0.60**	0.60**	0.54**	0.41**
Inappropriate speech	0.35*	0.42**	0.31*	0.34*

*Note*: *p <0.05. **p <0.001.

All sensory quadrants were significantly associated with lethargy, though demonstrated varying strengths of association: poor registration, seeking, and avoiding exhibited moderate relationships with this variable (*r =* 0.34, *p* <0.05; *r =* 0.41, *p* <0.001, and *r =* 0.48, *p* <0.001 respectively), while sensitivity was strongly associated with lethargy (*r =* 0.58, *p* <0.001). Stereotypic behavior also significantly correlated with all quadrants, with seeking (*r =* 0.42, *p* <0.001), and sensitivity (*r =* 0.43, *p* <0.001) displaying the strongest (though moderate) associations. Furthermore, inappropriate speech was also significantly associated with all quadrants, with avoiding exhibiting the strongest (though moderate) relationship (*r =* 0.42, *p* <0.001).

### Associations between sensory processing and caregiver strain

To address our second aim, Pearson's correlations were performed to examine the differential relationships between sensory quadrants and caregiver strain. Looking first at global scores on the CGSQ, all sensory quadrants were significantly and at least moderately correlated with overall caregiver strain, with avoiding (*r =* 0.71, *p* <0.001) and sensitivity (*r =* 0.61, *p* <0.001) displaying strong associations, as shown in Table 3. Within domain scores on the CGSQ, avoiding demonstrated the strongest positive relationship with objective strain (*r =* 0.75, *p* <0.001), while sensitivity (*r =* 0.66, *p* <0.05), seeking (*r =* 0.54, *p* <0.001) and poorer registration (*r =* 0.49, *p* <0.001) were also significantly correlated with objective strain. Avoiding also demonstrated a moderate association with subjective internalized and externalized strain (*r =* 0.48 and *r =* 0.44 respectively, *p* <0.001). Sensitivity also exhibited a significant and moderate positive relationship with subjective internalized strain (*r =* 0.42, *p* <0.001).

**TABLE 3:** Pearson's correlations between sensory quadrants and caregiver strain

	Seeking	Avoiding	Sensitivity	Registration
Objective strain	0.54**	0.75**	0.66**	0.49**
Subjective internalized strain	0.31*	0.48**	0.42**	0.27*
Subjective externalized strain	0.26*	0.44**	0.25*	0.30*
Global score	0.49**	0.71**	0.61**	0.46**

*Note*: *p <0.05. **p <0.001.

### Sensory processing and maladaptive behaviors: Predicting caregiver strain

The third aim of our study was to determine how maladaptive behaviors and atypical sensory processing features predict caregiver strain and examine the individual contribution of each sensory processing quadrant to this relationship. A multiple regression was performed with maladaptive behaviors and sensory processing as predictors of global caregiver strain.

The assumptions for a hierarchical multiple regression were tested prior to conducting this statistical test. Linearity was assessed by plotting studentized residuals against predicted values and by looking at partial regression plots. Similarly, homoscedasticity was assessed through a visual inspection of the plot of studentized residuals against unstandardized predicted values. There was independence of residuals, as assessed by the Durbin‐Watson statistic of 1.75, and no evidence of multicollinearity based on Tolerance/VIF values. No cases had studentized deleted residuals greater than ±3 *SD* and no cases exerted undue influenced over the model as evidence by leverage values and Cook's distance values in acceptable ranges. Finally, Q‐Q plots were assessed to verify the assumption of normality.

A 2‐step hierarchical multiple regression was conducted with global caregiver strain as the dependent variable. The maladaptive behavior domains of the ABC‐P were entered at step one (i.e., inappropriate speech, stereotypic behavior, irritability, hyperactivity/noncompliance, lethargy/social withdrawal), and SSP‐2 domain scores (avoiding, seeking, sensitivity and registration) were entered at step two. The variables were entered in this order as we sought to observe how much additional variance in global caregiver strain was explained by sensory processing, above that explained by maladaptive behaviors. Intercorrelations between the multiple regression variables are reported in Table S2 and the regression statistics are displayed in Table 4.

**TABLE 4:** Summary of hierarchical regression analysis for variables predicting global Strain

Variable	*β*	t	$sr^{2}$	*R*	$R^{2}$	∆$R^{2}$
Step 1				0.63*	0.40*	
Irritability	0.41	2.90*	0.073			
Lethargy/withdrawal	0.12	0.82	0.006			
Stereotypic behavior	0.03	0.23	0.000			
Hyperactivity	0.14	1.06	0.010			
Inappropriate speech	0.03	0.27	0.001			
Step 2				0.76*	0.58*	0.18*
Irritability	0.20	1.39	0.013			
Lethargy/withdrawal	−0.10	−0.73	0.003			
Stereotypic behavior	0.20	1.55	0.016			
Hyperactivity	−0.02	−0.13	0.000			
Inappropriate speech	0.00	0.00	0.000			
Sensory avoiding	0.45	3.02*	0.059			
Sensory seeking	−0.18	−1.09	0.008			
Sensory sensitivity	0.24	1.52	0.015			
Low registration	0.15	1.06	0.007			

*Note*:. ∆$R^{2}$ = change in *R* ^2^ from Step 1 to Step 2. sr^2^ = squared semi partial correlation coefficient. *p <0.01.

Introducing the variables pertaining to maladaptive behaviors at Step 1 showed that they contributed significantly to the regression model, F (5,69) = 9.27, *p* <0.0001, accounting for 40% of the variance in global caregiver strain. Furthermore, irritability alone was a unique predictor of global caregiver strain at this step, uniquely explaining 7.3% of variation in caregiver strain (*p* =0.005). Introducing the sensory processing variables at Step 2 explained an additional 18% of the variance in global caregiver strain. When all 9 independent variables were included at Step 2, the only significant predictor of global caregiver strain was sensory avoiding, which uniquely explained 5.9% of the variation (*p* =0.004). Together, the nine independent variables accounted for 57.9% of the variance in global caregiver strain, F (9, 65) = 9.94, *p* <0.001.

## DISCUSSION

Our findings suggest that the four sensory quadrants of Dunn's model (sensitivity, seeking, avoiding, and registration) are differentially associated with maladaptive behaviors, as indexed by scores on the ABC‐P. Secondly, our results revealed distinct relationships between atypical sensory processing features and caregiver strain, as indexed by the SSP‐2 and CGSQ. Thirdly, we found that together, maladaptive behaviors and sensory processing (specifically sensory avoiding) were able to account for 57.9% of the variance in global caregiver strain.

We found that atypical sensory features were positively and significantly associated with maladaptive behavior domains across the ABC‐P, consistent with prior findings (Baker et al., 2008; Lane et al., 2010; Nieto et al., 2017). Notably, maladaptive behaviors were differentially associated with sensory features, with large correlations observed between irritability and sensory avoiding, between lethargy and sensory sensitivity, and between hyperactivity, sensory seeking, sensory avoiding, and sensory sensitivity. Taken together, not only do these findings align with and extend the current literature (e.g., Dellapiazza et al., 2020), but they also have practical implications. Firstly, the results facilitate a more nuanced understanding of children's “maladaptive behaviors” and the potential triggers causing these behaviors (e.g., a child's hyperactivity may serve a function of avoiding aversive stimuli, or their agitation a consequence of attempts to continuously monitor their environment to avoid sensory distress). Therapeutic supports targeted at reducing sensory avoiding features may also reduce hyperactivity, irritability, and agitation.

Our results also support previous findings that atypical sensory processing is related to degree of caregiver strain, suggesting that as sensory processing difficulties increase, so too does caregiver stress (Gourley et al., 2013; Nieto et al., 2017). Sensory avoiding appeared to be most strongly associated with caregiver strain. This finding is consistent with that of Nieto et al. (2017), who found that sensory avoiding was significantly and most strongly related to caregiver distress. Our study also found a strong association between sensory sensitivity and global caregiver strain. To further clarify these relationships, we then examined how maladaptive behaviors, and atypical sensory processing features together predicted overall caregiver strain. We found that maladaptive behaviors alone accounted for 40.2% of the variance in caregiver strain. Interestingly, the only domain of the ABC‐P that significantly contributed to variance in caregiver strain was irritability. This aligns with recent findings from Bradshaw et al. (2021), where disruptive, externalizing behaviors such as irritability predicted caregiver strain. When sensory processing features were added into the final step of the regression, the model accounted for 58% of the variance in overall caregiver strain. When all variables pertaining to maladaptive behaviors and sensory processing were included in our regression model, the only significant predictor of overall caregiver strain was sensory avoiding, which uniquely accounted for 5.8% of this variance.

Overall implications for this finding with respect to caregiver well‐being are twofold. Firstly, it may be useful to predict which caregivers of children with autism are at the highest risk of increased strain, so health professionals are prepared to offer assistance to prevent depression and its associated outcomes (Bromley et al., 2004). In this vein, Bradshaw et al. (2021) highlighted that a nuanced understanding of how caregiver strain is uniquely associated with a child's behavior difficulties, and internalizing and externalizing symptoms, can guide targeted support. Secondly, it may be of value to explore the efficacy of sensory modifications and strategies in reducing caregiver strain. While current sensory interventions such as sensory integration therapy have demonstrated some promising results (Pfeiffer et al., 2011) the current study provides impetus for further development of strategies targeting specific sensory features. Our results provide a preliminary rationale for exploring sensory‐based interventions on caregiver strain and, in turn, how this may impact parenting, quality of life, and family wellbeing. It may be of value to explore the effect of modifying the family home or classroom environment to be suited to individualized sensory needs, and to explore whether this yields improvements in behavioral problems and, in turn, caregiver strain. Behavioral interventions such as the Early Denver Model, which focuses on social attention, affect sharing imitation, joint attention, and positive behavioral supports, have also been shown to decrease behaviors of concern (Fulton et al., 2014). It may be of interest to future researchers to explore whether such interventions could also play a role in supporting sensory difficulties and perhaps in turn, target parental strain.

A limitation of much of the research in this area, including the present study, is the reliance on caregiver‐report questionnaire measures to index sensory processing, maladaptive behaviors, and caregiver strain. Future studies would benefit from utilizing these measures in combination with objective outcome measures such as researcher observation of child behaviors or reaction to sensory stimuli. Given our sample size, we acknowledge that our findings are preliminary. Further investigation with larger samples and increased statistical power is warranted, to confirm and extend our results. Additionally, as is the case with the much of the research on autism, our sample consisted of relatively few girls, and may be less reflective of sensory processing or its relationship with other outcomes in females. In contrast to recent findings from (Osório et al., 2021), we did not observe any sex differences in sensory processing, however, this may be due to the small number of females in our sample. Increasing evidence suggests that females on the autism spectrum can display substantively different sensory processing features to their male peers (Kozlowski et al., 2012; Osório et al., 2021; Van Wijngaarden‐Cremers et al., 2014). Further, recent findings suggest that there are sex differences in the neural mechanisms underlying sensory over‐responsivity (Cummings et al., 2020), suggesting that future research would benefit from further examination of sex‐related differences in sensory symptoms.

Overall, we found distinct relationships between sensory quadrants, maladaptive behaviors, and caregiver strain, which have important clinical implications for both child and caregiver outcomes. Studies further clarifying these relationships using a validated measure of maladaptive behaviors, such as the ABC‐P, are warranted, as well as refinement of specific sensory interventions. Our study highlights the utility of identifying specific sensory processing features, in addition to maladaptive behaviors, to inform on interventions, and potentially decrease caregiver strain.

## CONFLICT OF INTEREST

Professor Ian Hickie was an inaugural Commissioner on Australia's National Mental Health Commission (2012–2018). He is the Co‐Director, Health and Policy at the Brain and Mind Centre (BMC) University of Sydney. The BMC operates an early‐intervention youth services at Camperdown under contract to headspace. Professor Hickie has previously led community‐based and pharmaceutical industry‐supported (Wyeth, Eli Lily, Servier, Pfizer, AstraZeneca) projects focused on the identification and better management of anxiety and depression. He was a member of the Medical Advisory Panel for Medibank Private until October 2017, a Board Member of Psychosis Australia Trust and a member of Veterans Mental Health Clinical Reference group. He is the Chief Scientific Advisor to, and a 5% equity shareholder in, InnoWell Pty Ltd. InnoWell was formed by the University of Sydney (45% equity) and PwC (Australia; 45% equity) to deliver the $30 M Australian Government funded Project Synergy (2017–2020; a three‐year program for the transformation of mental health services) and to lead transformation of mental health services internationally through the use of innovative technologies.

## Supporting information


**Supplementary Table 1** Correlations between IQ, age, Caregiver Strain, Sensory features, and challenging behaviors
**Supplementary Table 2.** Intercorrelations between regression variables

## Data Availability

The data that support the findings of this study are available from the corresponding author upon reasonable request.
