# Supplementary material for: Atypical sensory processing features in children with autism, and their relationships with maladaptive behaviors and caregiver strain
Source: Autism Res. 2022 Mar 17;15(6):1120–9. doi: 10.1002/aur.2700 (PMC9544661; doi:10.1002/aur.2700)
Supplement: Supplementary file 1 — Supplementary Table 1 Correlations between IQ, age, Caregiver Strain, Sensory features, and challenging behaviors Supplementary Table 2. Intercorrelations between regression variables [file AUR-15-1120-s001.docx]

**Supplementary Table 1.**

*Correlations between IQ, age, Caregiver Strain, Sensory features, and challenging behaviours*

|  | Age | Leiter 3 FSIQ |
| --- | --- | --- |
| GGS – Global score | -.03 (*.810*) | -.04 (*.745*) |
| ABC – Irritability | .08 (*.524*) | -.14 (*.231*) |
| ABC – Lethargy/social withdrawal | .06 (*.586*) | -.11 (*.367*) |
| ABC – Stereotypic Behaviour | .03 (*.812*) | -.21 (*.074*) |
| ABC – Hyperactivity/non-compliance | -.15 (*.192*) | -.05 (*.686*) |
| ABC – Inappropriate Speech | -.11 (*.372*) | -.01 (*.922*) |
| SSP-2 – Sensory Seeking | -.19 (*.099*) | .07 (*.535*) |
| SSP-2 – Sensory Avoiding | -.11 (*.370*) | -.02 (*.900*) |
| SSP-2 – Sensory Sensitivity | -.01 (*.982*) | -.03 (*.834*) |
| SSP-2 – Low Registration | -.16 (*.180*) | .16 (*.161*) |

*Note:* Data presented as r(p-value)

**Supplementary Table 2**

*Intercorrelations between regression variables*

|  | CGS Global score | ABC Irritability | ABC Lethargy | ABC Stereotypic behaviour | ABC Hyperactivity | ABC Inappropriate speech | SSP-2 Seeking | SSP-2 Avoiding | SSP-2 Sensitivity | SSP-2 Registration |
| --- | --- | --- | --- | --- | --- | --- | --- | --- | --- | --- |
| CGS  Global score | ­– |  |  |  |  |  |  |  |  |  |
| ABC  Irritability | .61** | – |  |  |  |  |  |  |  |  |
| ABC  Lethargy | .47** | .59** | – |  |  |  |  |  |  |  |
| ABC  Stereotypic behaviour | .40** | .49** | .71** | – |  |  |  |  |  |  |
| ABC Hyperactivity | .52** | .68** | .54** | .55** | – |  |  |  |  |  |
| ABC Inappropriate speech | .35* | .50** | .39* | .26* | .43** | – |  |  |  |  |
| SSP-2  Seeking | .49** | .44** | .41** | .42** | .60** | .35* | – |  |  |  |
| SSP-2  Avoiding | .71** | .70** | .48** | .29* | .60** | .42** | .60** | – |  |  |
| SSP-2 Sensitivity | .60** | .46** | .58** | .43** | .54** | .31* | .76** | .68** | – |  |
| SSP-2 Registration | .46** | .29* | .33* | .26* | .41** | .34* | .78** | .52** | .66** | – |
